# Agriculture and the promotion of insect pests: rice cultivation in river floodplains and malaria vectors in The Gambia

**DOI:** 10.1186/1475-2875-8-170

**Published:** 2009-07-27

**Authors:** Lamin BS Jarju, Ulrike Fillinger, Clare Green, Vasilis Louca, Silas Majambere, Steven W Lindsay

**Affiliations:** 1 National Malaria Control Programme, Banjul, the Gambia; 2 Disease Control & Vector Biology Unit, London School of Hygiene and Tropical Medicine, London, UK; 3 School of Biological and Biomedical Sciences, Durham University, Durham, UK; 4 Ifakara Health Institute, Dares Salaam, Tanzania

## Abstract

**Background:**

Anthropogenic modification of natural habitats can create conditions in which pest species associated with humans can thrive. In order to mitigate for these changes, it is necessary to determine which aspects of human management are associated with the promotion of those pests. *Anopheles gambiae*, the main Africa malaria vector, often breeds in rice fields. Here the impact of the ancient practice of 'swamp rice' cultivation, on the floodplains of the Gambia River, on the production of anopheline mosquitoes was investigated.

**Methods:**

Routine surveys were carried out along 500 m transects crossing rice fields from the landward edge of the floodplains to the river during the 2006 rainy season. Aquatic invertebrates were sampled using area samplers and emergence traps and fish sampled using nets. Semi-field experiments were used to investigate whether nutrients used for swamp rice cultivation affected mosquito larval abundance.

**Results:**

At the beginning of the rainy season rice is grown on the landward edge of the floodplain; the first area to flood with fresh water and one rich in cattle dung. Later, rice plants are transplanted close to the river, the last area to dry out on the floodplain. Nearly all larval and adult stages of malaria vectors were collected 0–100 m from the landward edge of the floodplains, where immature rice plants were grown. These paddies contained stagnant freshwater with high quantities of cattle faeces. Semi-field studies demonstrated that cattle faeces nearly doubled the number of anopheline larvae compared with untreated water.

**Conclusion:**

Swamp rice cultivation creates ideal breeding sites for malaria vectors. However, only those close to the landward edge harboured vectors. These sites were productive since they were large areas of standing freshwater, rich in nutrients, protected from fish, and situated close to human habitation, where egg-laying mosquitoes from the villages had short distances to fly. The traditional practice of 'swamp rice' cultivation uses different bodies of water on the floodplains to cultivate rice during the rainy season. A consequence of this cultivation is the provizion of ideal conditions for malaria vectors to thrive. As the demand for locally-produced rice grows, increased rice farming will generate great numbers of vectors; emphasizing the need to protect local communities against malaria.

## Background

It is ironic that the world's huge agro-ecosystems designed to feed the ever increasing human population also provide a habitat for a far greater number of insects to exploit and thrive in. Annually approximately 18% of the world's crops are damaged or consumed by insect pests [1]. Agro-ecosytems also provide ideal breeding habitats for many insects. For example, irrigation is associated with the production of vectors that transmit pathogens to humans, including those responsible for malaria [2]. In order to manage vector populations, it is important to know which specific human practices promote these pests.

It is well known that rice cultivation leads to increased mosquito production in Africa [3-7]. Increases in the number of *Anopheles gambiae sensu lato*, the major malaria vector, typically correspond with the beginning of rice cultivation, when paddies are first flooded and rice is short [5,8-11]. Most research on rice and malaria focuses on irrigated rice production [10-16] and rarely traditional practices [17,18].

Rice has been cultivated in The Gambia for many centuries [19]. Traditional lowland rice is grown in the floodplain of the Gambia River, and is known locally as '*bafaro*' or 'swamp rice'. Swamp rice production is one of the oldest forms of rice cultivation in West Africa, with approximately 200,000 ha under cultivation in Guinea, Guinea Bissau, Senegal, Sierra Leone and The Gambia [20]. During the rainy season in The Gambia, a combination of heavy rainfall and a rising river level results in major flooding. It is in the floodplain that the rice fields are constructed during the rainy season, between 110–290 km from the river mouth, where the river is tidal and brackish at certain times of the year [21]. Recent studies of larval habitats along the middle reaches of the river demonstrated that these rice fields are the most common aquatic habitat in this area [22]. Since rice fields often cover the entire floodplain we wanted to know whether there is a difference in the spatial distribution of larvae and, most importantly, production of adult vectors across the floodplain. This information is important since it may be possible to target interventions at particular sites. It was also of interest to determine whether nutrients applied to the rice fields affected the production of anophelines.

## Methods

### Study area

**Figure 1:**
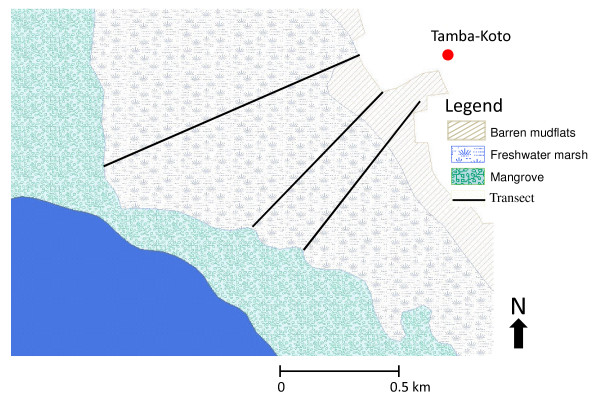
**Map of study area**.

The study was carried out near Tamba Koto (13° 31.776'N, 15°30.990 W), close to the Gambia River (Figure 1). The village had about 215 inhabitants, predominantly Mandinka. The area is generally flat, open farmland and sparse woodland, typical of Sudan savannah. The rainy season is from June to October, followed by a long dry season from November to May. The semi-field study was carried out at the Medical Research Council Field Station, in Farafenni town (15°00.200 N, 43°55.00 E), where rainfall data were also collected.

### Aquatic surveys

**Figure 2:**
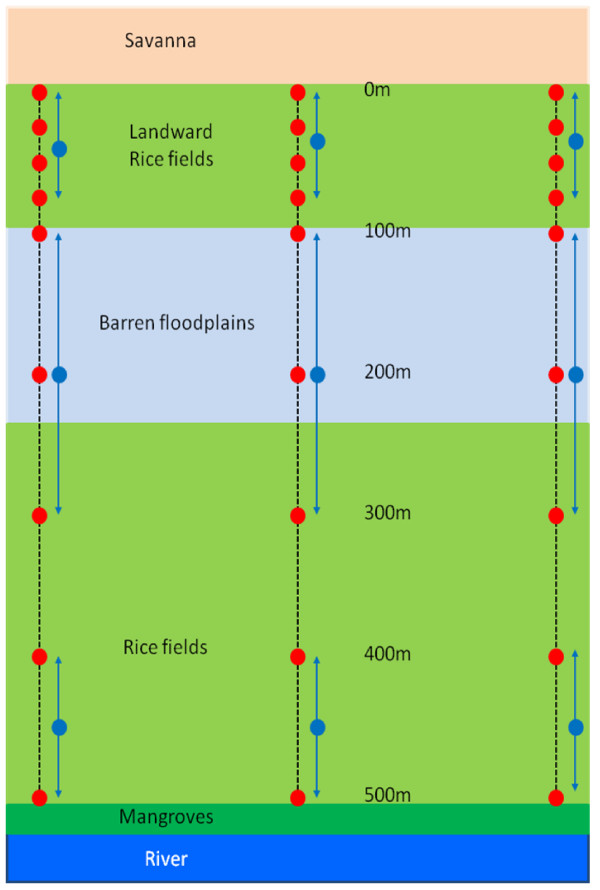
**Schematic representation of sampling frame in the study area**. Where broken lines represent the three transects, red circles represent weekly sampling points for aquatic invertebrates and blue circles weekly sampling points for emerging invertebrates.

Three parallel transects each approximately 500 m long, 20 m wide, and 200 m apart were sampled weekly from June 2006 to January 2007. Transects were situated across the floodplains of the Gambia River, starting on the landward edge near the village and ending near the river (Figure 1). Since mosquitoes were found breeding on the edge of the floodplains [23,24], sampling was concentrated here. Larvae were sampled at 0 m, 25 m, 50 m, 75 m, 100 m, 200 m, 300 m, 400 m and 500 m along each transect (Figure 2). At each distance three samples were made with an area sampler (AS; [25]), one at the centre and two at opposite ends of the respective paddy. The AS was a 39.5 cm long aluminium tube, with serrated teeth around the bottom lip to grip into the substrate (upper diameter = 47 cm, lower one = 40 cm; surface area of 0.126 m^2^). It was plunged quickly into water most likely containing larvae (i.e. edge of water or near emergent vegetation) and left for 30 seconds to allow water to settle and larvae to rise to the surface. A dipper (Clarke Mosquito Control Products, Illinois, USA) was used to empty water from the AS and transferred into a white plastic bowl containing clear water. Excess water was carefully removed to concentrate any organisms present, and the specimens placed in 100% ethanol and transported to the laboratory for identification.

### Emergence fauna

Floating emergence traps were used to sample adult insects [26-28], in three zones along each transect: 0–75 m from the landward edge, 100–300 m and 400–500 m at weekly intervals from June 2006 to January 2007 (Figure 2). Traps were positioned 4 m either side of each transect over water bodies thought likely to contain mosquitoes. In each zone samples were made: (1) <1 m from the field edge nearest the village, (2) in the centre of the field, and (3) in the same field near the edge furthest from the village. This procedure was repeated in all zones along each transect. Emergence traps were conical and constructed from metal rods. They were 1 m high, 1 m in diameter (0.786 m^2 ^surface area) and covered in transparent nylon netting [29]. Traps were made buoyant by attaching plastic 1 L bottles to the base to allow the water to flow undisturbed under the trap and therefore allow aquatic organisms, including potential insect predators, to move freely in and out of the trap. Each trap was tethered to the ground allowing the trap to rotate freely. The top of each cone opened into a collection chamber (Bioform, Germany), filled with 250 ml of 60% glycol to kill and preserve flying insects that collected there. A netting sleeve on the side of each trap allowed flying insects to be collected with an aspirator. Insects were removed weekly and transported to the laboratory for identification. Traps were moved every week to a new location within the same sampling area, where larvae had recently been found.

### Specimen identification

All insects, excluding mosquitoes, were separated into the following taxonomic groups: broad-shouldered water striders (Veliidae), beetle adults (Coleoptera), beetle larvae (Coleoptera), creeping water bug adults (Noucoridae), damselfly larvae (Odonata; sub-order Zygoptera), dragonfly larvae (Odonata; sub-order Anisoptera), greater water boatman adults (Notonectidae), lesser water boatman adults (Corixidae), mayfly larvae (Ephemeroptera), pigmy backswimmer adults (Pleidae), pond skater adults (Gerridae), water measurer adults (Hydrometridae) and water scorpion adults (Nepidae). Mosquito larvae were counted and identified as anophelines or culicines in the field. Adult anopheline mosquitoes were identified with morphological keys [30] and members of the *An. gambiae *complex identified by PCR analysis [31].

### Fish sampling

Fish were sampled using a cast net (diameter: 230 cm, mesh size: 10 mm) and a hand net (25 × 17 cm, mesh: 2 mm) at 50 m intervals, as in the larval sampling, once along each transect in August. At each sampling point three cast-net throws were made at different locations within 10 m of either side of the transect point. Five cumulative minutes of sweeping were also undertaken with the hand-net within the same sampling area, with only enough time between sweeps and net throws to remove the fish from the net. Fish were identified to species using morphological keys [32].

### Physical measurements

At each mosquito survey point the following information was recorded. Water depth was measured with a metre rule at three different locations and averaged. The presence of tidal water was recorded visually. Water conductivity, pH, temperature and dissolved oxygen content were measured with a multi-parameter probe (350i WTW, Germany) and water turbidity with a turbidity meter (HANNA, USA). Samples were taken between 07:00–14:00 h. Rice height was measured from the water surface to the maximum vertical height of the plant. Counts of cow dung were made in a 200 m^2 ^area (20 m wide and 10 m long) at 0 m, 25 m, 50 m, 75 m, 100 m, 200 m, 300 m, 400 m and 500 m along each transect in June, before the rains.

### Semi-field experiments

Whether nutrients commonly found in rice fields affected mosquito production was investigated. Nine mauve plastic bowls filled with 20 L of tap water served as mosquito breeding sites (surface area of 0.21 m^2^). Bowls were arranged in open grassland in a 3 × 4 grid, with each bowl 1 m from its neighbour. Approximately 5 g of floodplain soil was added to each bowl in order to provide conditions suitable for mosquitoes [33,34].

Each bowl had one of the following treatments: cow dung (20 g), urea (200 g, 46% nitrogen, Honorich Technology Co., China), with tap water serving as a control. The amount of cow dung and urea approximated that seen in the field. Treatments were allocated to bowls in a balanced design. For each of the four trials, each treatment was randomly allocated a different bowl number. Bowls were covered for two days to allow the soil to settle and then left open for wild mosquitoes to lay their eggs for seven days. Larval sampling was done using a 350 ml dipper: five scoops were taken from the surface of each bowl, four on the edges and one in the centre. This was done daily for 14 days in each trial. Mosquito larvae were counted and returned to the bowl. 1^st ^and 2^nd ^stage larvae were recorded as early instars and 3^rd ^and 4^th ^stage larvae as late instars. Pupae were removed from the bowls, counted and transferred into separate cages for each treatment where adult mosquitoes emerged. All mosquitoes were identified as described earlier.

### Nutrient analysis

Nutrients were measured in nine bowls, each containing the three different treatments (i.e. 3 bowls/treatment) and sampled after 1, 4 and 7 days. 250 ml water samples were filtered using 0.45 μm cellulose acetate membrane filters and analysed spectrophotometrically for filterable reactive phosphorus (FRP-PO_4_^3-^) following the ascorbic acid method [35], for filterable reactive nitrogen (FRN-NO_3_/NO_2_) using the Feree method [36] and ammonia (NH_4_), following the Nessler method [35]. Colour, an indirect measure of the concentration of tannins, was measured at 440 nm [37]. All measurements were carried out within 3 h of collection to minimize any change in water quality over time.

### Statistical analysis

Non-normal data were normalized by log transformation or squared. Comparisons between normally distributed data were made using t-tests and between proportions using chi-square analysis. All variables were incorporated untransformed in a mathematical model and their overall impact on the presence or absence of anopheline larvae or adults explored using Generalized Estimating Equations (GEE). This was a logistic model and adjusted for repeated measures. GEE were also used to examine the relationship between larval numbers and treatment group. Analyses were performed with SPSS version 15. Missing data and data from emergence traps that were not fully working were excluded from the analysis.

### Ethical approval

Approval for this study was given by the Joint Gambian Government and Medical Research Councils Laboratories, The Gambia, Ethics Committee and the Ethics Advisory Committee of Durham University. Village meetings were held with village elders and women groups to explain the purpose of this study and to gain approval for the work.

## Results

### Meteorology

Total rainfall during the 2006 rainy season was 807.9 mm. Rain started in the beginning of June and ended in the middle of October. The rainfall was similar to the mean annual rainfall of 772.8 mm (95% Confidence intervals = 694.9–850.7 mm) for the period 1990–2005.

### Flooding patterns in the river floodplains

The landscape from Tamba Koto to the River Gambia is characterized by upland agricultural fields, the tree-lined fringe of upland savannah, followed by barren mud, before the first rice fields and large areas of floodwater beyond. Further into the floodplain tall reeds are found before reaching the second area of rice fields close to the river and the mangrove forest fringing the banks of the river. Different parts of the floodplain experience different patterns of flooding (Table 1). Rice fields 0–100 m from the landward edge flooded in August, filled with rain water. In September the rice fields close to the river were flooded due to a combination of rainfall and rising river level. Whilst paddies close to land dried out by the middle of October, those near the river were more permanent and did not completely dry until January.

**Table 1:** Seasonality of flooding in study area.

Month	Week	Distance from landward edge (m)								
		0	25	50	75	100	200	300	400	500
Jul	1									
	2									
	3									
	4									
Aug	1									
	2	XX	XX	XX	XX	XX				
	3	XX	XX	XX	XX					
	4	XX	XX	XX	XX	XX				
Sep	1	XX	XX	XX	XX		XX	XX	XX	XX
	2	XX	XX	XX	XX	XX	XX	XX	XX	XX
	3	XX	XX	XX	XX	XX	XX	XX	XX	XX
	4	XX	XX	XX	XX	XX	XX	XX	XX	XX
Oct	1	XX	XX	XX	XX	XX	XX	XX	XX	XX
	2	XX	XX	XX	XX	XX	XX	XX	XX	XX
	3				XX		XX	XX	XX	XX
	4	no data collection								
	5						XX	XX	XX	XX
Nov	1				X		XX	XX	XX	XX
	2						XX	XX	XX	XX
	3						XX	XX		
	4						XX	XX	XX	XX
Dec	1						XX	XX	XX	XX
	2							XX	XX	XX
	3							XX	XX	XX
	4							XX	XX	XX
Jan	1									XX
	2									XX
	3									
	4									
	5									

Crosses represents flooding in at least one of the three transects each week.

### Rice cultivation

Fields closer to the upland were divided into small fields with high embankments built to help conserve rain water, whilst those nearer the river were less clearly demarcated and subject to flooding by tidal water. Rice cultivation started in June when farmers ploughed their fields. 'Nerica' rice was sown twice on raised nursery beds close to the landward edge of the alluvial floodplains. The first seedlings were transferred to the fields closest to the landward edge by the end of August. Here the rice was grown to maturity, even though some fields were not water-logged later in the season. The second batch of seeds were sown in the nursery beds in early August and transplanted to the fields near the river from late September to the end of October. Urea was applied to fields by hand when transplanting rice plants in September or shortly afterwards at a dose of 25 Kg of urea to 50 m^2^. Rice was harvested from December to January, starting with paddies near the landward edge. No insecticides were applied to the fields.

### Physical measurements

Water in the fields on the landward edge of the floodplain was stagnant, shallower, warmer, with a lower conductivity and pH and richer in cow dung (80% of deposits) than water in fields close to the river (Table 2).

**Table 2:** Characteristics of water and distribution of invertebrates along the transects obtained during larval surveys.

Variables	Distance from landward edge		P
	0–100 m n = 140	200–500 m n = 415	
^a^Depth (cm)	9.0 (8.4–9.7)	10.7 (10.1–11.2)	0.001
^a^Turbidity (ntu)	107.9 (86.9–133.9)	129.0 (120.8–137.8)	ns
^b^pH	7.0 (6.8–7.3)	7.6 (7.5–7.7)	<0.001
^a^Conductivity (mS/cm)	1.0 (0.9–1.2)	2.5 (2.3–2.7)	<0.001
^a^Temperature°C	30.2 (29.8–30.6)	28.6 (28.3–28.9)	<0.001
Oxygen content units (mg/L)	5.6 (5.2–6.1)	6.0 (5.8–6.2)	ns
Presence of moving water (%)	4.3%	99.0%	<0.001
Height of rice (cm)	17.0 (12.9–21.1)	39.1 (35.5–42.7)	<0.001
Cow dung samples/site	132	33	<0.001
	N = 638	N = 802	
Total anopheline larvae (proportion of occasions trapped)	349 (58/638)	26 (13/789)	<0.001
*Anopheles gambiae s.l*.	66 (19/638)	14 (9/802)	0.011
*Anopheles gambiae s.s*.	15 (3/638)	8 (2/802)	ns
*Anopheles arabiensis*	30 (6/638)	6 (2/802)	ns
*Anopheles melas*	21 (4/638)	0 (0/802)	0.038
Culicine larvae	423 (53/638)	19 (7/802)	<0.001
Other aquatic insects	912 (19/638)	532 (9/802)	<0.001
Mean no. invertebrate taxa, excluding mosquitoes	3.1 (2.6–3.6)	0.9 (0.8–1.0)	<0.001
Mean no. fish species/sample	0	1.19 (0.68–1.59)	

Values shown are means after the data were normalized ^a ^by log transformation (ln(x +1)), or ^b ^by squaring values. Figures in parenthesis represent 95% confidence intervals for abiotic variables and proportion of sites with specimens for biotic variables.

### Aquatic invertebrates

**Figure 3:**
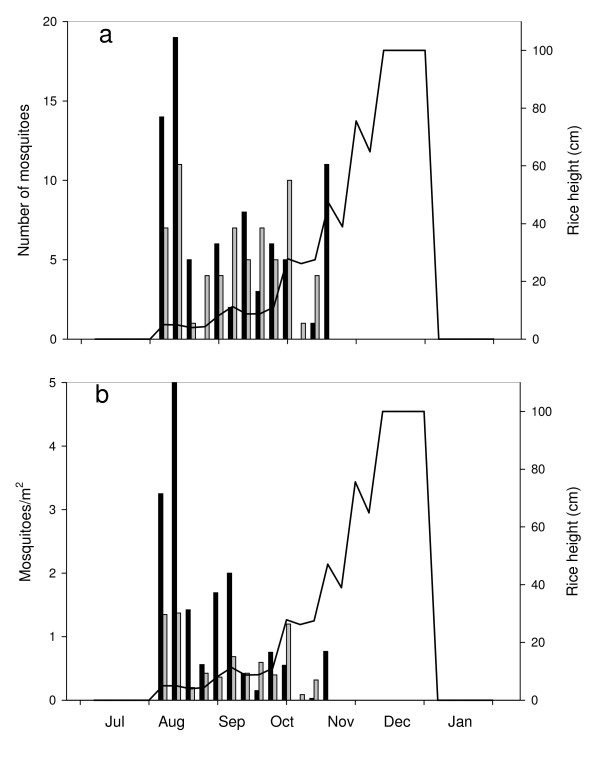
**Seasonality of anopheline larvae and adults in ricefields**. a is the total number of anophelines collected, whilst b is anopheline density. Where black bars are weekly larval collections, grey bars are total number of adults collected weekly and the solid line is the average height of rice.

375 anopheline larvae and 442 culicine larvae were collected from 555 samples. There were 80 *An. gambiae s.l*. of which 45% were *Anopheles arabiensis*, 29% *An. gambiae s.s*. and 26% *Anopheles melas*; equivalent to 1.14 *An. gambiae s.l*./m^2^. Members of the *An. gambiae *complex were found shortly after the fields were first flooded in August (Figure 3), but their numbers fell to zero in early November coincident with the drying out of the fields close to the landward edge (Table 1) and increased height of rice. Most aquatic invertebrates were sampled 0–100 m from the landward edge of the alluvial floodplains (i.e. 83% anophelines, 96% culicines and 63% of other invertebrates). Even though the first 100 m of each transect were sampled more intensively than sites further away, the sites closer to land dried out more quickly. Thus, the sites 0–100 m from the landward edge were wet on 164 occasions, compared with 391 occasions in sites further away. After adjusting for differing sampling effort, by calculating the mean number of specimens collected on each occasion in different parts of the floodplain, 92% of *An. gambiae s.l*. larvae were found in the first 100 m of each transect.

Multivariate modelling revealed that the presence of all anophelines and *An. gambiae s.l*. larvae was highest within rice fields 0–100 m from the landward edge of the floodplains (Table 3). Within each paddy larvae were more common along the edge than the centre. The presence of short rice plants in area samplers and culicine larvae were also positively associated with the presence of anopheline larvae and those of *An. gambiae s.l*. The abundance of *An. gambiae s.l*. larvae also increased with the number of insect taxa (r^2 ^= 0.19, F = 123.5, P < 0.001).

**Table 3:** Multivariable analysis: factors associated with the presence or absence of anopheline larvae.

Variables	All anophelines				*An. gambiae s.l*.			
	Wald	Odds Ratio (OR)	95% OR	*P*	Wald	Odds Ratio (OR)	95% OR	*P*
***Spatial measurements***								
Distance from landward edge of transect								
1–100 m		1.00				1.00		
200–500 m	40.5	0.04	0.01–0.10	<0.001	13.15	0.13	0.04–0.39	<0.001
Position of sampling point within rice field								
Landward edge		1.00				1.00		
Centre	4.97	0.23	0.06–0.84	0.026	5.75	0.10	0.02–0.66	0.016
Riverside edge	3.95	0.31	0.10–0.98	0.047	2.42	0.33	0.08–1.34	0.081
***Biotic measurements***								
Rice in area samples								
Absent		1.00				1.00		
Present	13.78	28.25	4.84–164.85	<0.001	7.01	10.53	1.84–60.16	0.008
Culicine larvae								
Absent		1.00				1.00		
Present	9.72	2.78	1.46–5.30	0.002	14.23	6.06	2.38–15.44	<0.001
Insect biodiversity								
	2.92	2.16	0.89–5.20	0.087	0.17	1.21	0.50–2.95	ns

### Mosquito adult emergence

90 anopheline and 140 culicine adults were collected from 279 samples made with emergence traps (Table 4). There were 66 *An. gambiae s.l*. of which 92% were *An. arabiensis*, 6% *An. gambiae s.s*. and 2% *An. melas*; equivalent to 0.30 *An. gambiae *s.l./m^2^. *An. gambiae s.l*. were collected when the rice fields were first flooded in August (Figure 3), but the last adult was collected in early November coincident with the drying out of the fields close to the landward edge (Table 1) and increased height of rice. Most invertebrates were also collected 0–100 m from the landward edge (i.e. 94% *An. gambiae s.l*., 100% of other anophelines, 95% culicines and 60% of other invertebrates) even though nearly twice as many samples were made elsewhere (i.e. 101 wet sites 0–100 m from landward edge compared with 187 wet sites sampled further away). After adjusting for differences in sampling effort in different parts of each transect, 97% of *An. gambiae s.l*. adults were found in the first 100 m of each transect. The water within the first 100 m of each transect was shallower, non-tidal and had smaller, and therefore younger, rice plants compared with the fields further away which were characterized by deeper, tidal water where taller, more mature rice plants were transplanted.

**Table 4:** Characteristics of water parameters and distribution of adult mosquitoes and invertebrates along the transects.

Variables	Distance from landward edge		P
	0–100 m n = 91	200–500 m n = 188	
^a^Depth (cm)	9.80 (9.2–10.5)	11.9 (10.3–11.5)	0.032
Presence of moving water	10/91	188/188	<0.001
Height of rice (cm)	17.0 (12.9–21.1)	39.1 (35.5–42.7)	<0.001
*Anopheles gambiae s.l*.	62(36/91)	4 (3/188)	<0.001
*Anopheles gambiae s.s*.	4(4/91)	0(0/188)	0.001
*Anopheles arabiensis*	57(34/91)	4(3/188)	<0.001
*Anopheles melas*	1(1/91)	0(0/188)	ns
Other anophelines	24 (12/91)	0 (0/188)	<0.001
Culicine adults	133 (49/91)	7 (6/188)	<0.001
Other aquatic insects	353 (69/91)	234 (88/188)	<0.001
Insect families	2.2 0(1.8–2.5)	0.9 (0.7–1.0)	<0.001

Values shown are mean values after the data were normalized ^a ^by log transformation (ln(x +1)). Figures in parenthesis represent 95% confidence intervals for abiotic variables and proportion of sites with specimens for biotic variables.

**Figure 4:**
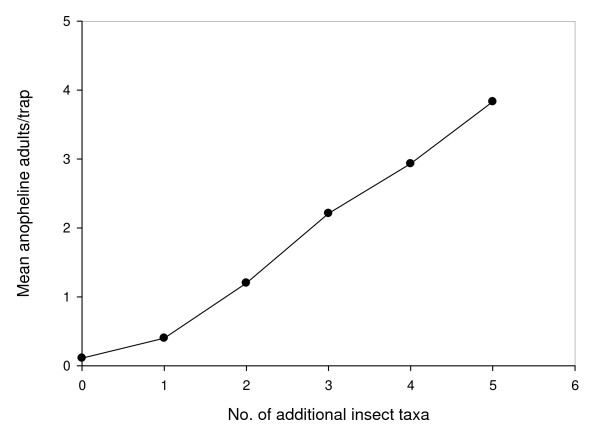
**Relationship between anopheline mosquitoes and diversity of other emergent insects**.

Multivariate modelling demonstrated that adult *An. gambiae s.l*. were more common in rice fields 0–100 m from the landward edge of the floodplains, particularly along the field edges (Table 5). Emergence of anopheline adults was associated with shorter rice plants and the simultaneous emergence of culicine adults. Whilst there was a strong relationship between the abundance of *An. gambiae s.l*. adults and insect richness (Figure 4; r^2 ^= 0.43, F = 62.2 P < 0.001), after adjuting for covariates the number of invertebrate taxa was of borderline statistical significance, suggesting that *An. gambiae s.l*. adult emergence was weakly associated with higher invertebrate diversity.

**Table 5:** Multiple variable analyses: factors associated with anopheline adult emergence.

Variable		*An. gambiae s.l*.		
	Wald	Odds Ratio (OR)	95% CIs OR	*P*
***Spatial measurements***				
Distance from landward edge of transect				
1–100 m		1.0		
200–500 m	4.1	0.23	0.05–0.95	0.042
Position of sampling point within rice field				
Edge		1.0		
Centre	4.9	0.23	0.06–0.84	0.026
***Biotic measurements***				
Height of rice (cm)				
	3.4	0.99	0.98–1.00	0.024
Culicine adults				
	36.8	2.22	1.72–2.88	<0.001
Insect richness				
	3.4	1.78	0.97–3.28	0.065

### Fish sampling

Four species of fish were collected: 17 *Periophthalmus barbarus*, 12 *Tilapia guineensis*, 1 *Epiplatys spilargyreius *and 1 *Porogobius schlegelli*. No fish were collected 0–100 m from the landward edge of each transect, whilst 1.19 species of fish were caught on average every sampling occasion at more distant sites (Table 2).

### Semi-field trials

A total of 6,233 anophelines and 11,234 culicine were collected during the four trials. Of the 135 members of the *An. gambiae s.l *complex collected 55% were *An. arabiensis *(n = 74), 44% *An. gambiae s.s*. (n = 59) and 1% *An. melas *(n = 2). Multivariate modelling revealed that the presence of cow dung in water significantly increased the number of anopheline and culicine larvae (Tables 6 and 7).

**Table 6:** Mean number of larvae per bowl and treatment.

	Mean number of larvae/bowl in different treatment (95% CI)		
	Untreated control	Urea	Cow dung
***Anopheline***			
Early instars	4.75 (2.85–7.93)	4.15 (2.36–7.28)	7.67 (4.97–11.83)
Late instars	0.92 (0.48–1.79)	0.59 (0.28–1.23)	1.77 (1.11–2.81)
***Culicine***			
Early instars	6.84 (4.62–10.12)	5.63 (4.03–7.85)	11.94 (8.30–17.19)
Late instars	1.48 (0.89–2.46)	2.25 (1.35–3.74)	5.76 (3.57–9.31)

**Table 7:** GEE analyses of different treatments on larval density.

	**Anopheline larvae**				**Culicine larvae**			
	**Early instars**		**Late Instars**		**Early instars**		**Late Instars**	
**Explanatory variables**	**Odds Ratio (95% CI)**	***p***	**Odds Ratio (95% CI)**	***p***	**Odds Ratio (95% CI)**	***p***	**Odds Ratio (95% CI)**	***p***
**Replicate**								
1	1.00		1.00		1.00		1.00	
2	15.82 (8.30–30.15)	<0.001	3.91 (1.75–8.72)	<0.001	2.61 (1.61–4.25)	<0.001	1.39 (0.64–3.01)	0.400
3	14.53 (7.80–27.05)	<0.001	0.83 (0.36–1.91)	0.661	4.27 (2.88–6.34)	<0.001	0.78 (0.38–1.60)	0.498
4	1.47 (0.59–3.64)	0.405	0.96 (0.25–3.70)	0.956	2.37 (1.63–3.46)	<0.001	1.20 (0.49–2.89)	0.691
**Treatment**								
Water	1.00		1.00		1.00		1.00	
Urea	0.96 (0.56–1.67)	0.899	0.631 (0.30–1.35)	0.234	0.72 (0.48–1.07)	0.101	1.57 (0.78–3.13)	0.203
Cow dung	1.73 (1.20–2.50)	0.003	2.38 (1.12–5.09)	0.025	1.49 (0.97–2.28)	0.068	4.11 (2.17–7.78)	<0.001

**Figure 5:**
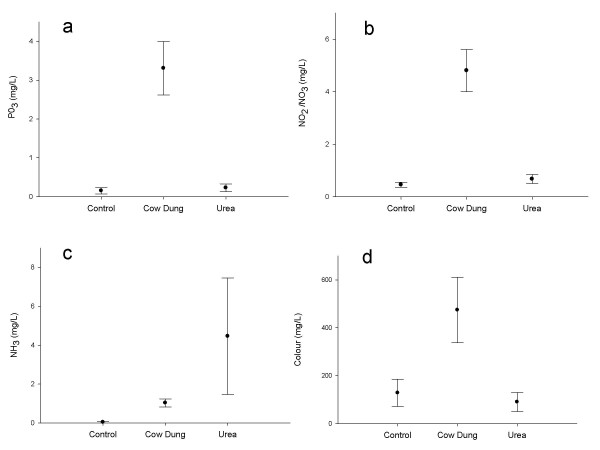
**Means and 95% C.I.'s of (a) FRP, (b) FRN, (c) NH3 and (d) colour for the different treatments**.

FRN, FRP, NH_4 _and colour differed significantly between treatments (Figure 5). There was 80% more nitrogen, 76% more phosphorous and 33% more NH_4 _in the cattle dung treatment compared to the control. Urea contained 76% more NH_4 _than the control. The water had 97% higher tannin content in the cattle dung treatment and 13% less in the fertilizer treatment (borderline significance) compared to the control.

## Discussion

This study demonstrates how humans exploit the changing conditions on the floodplain of the Gambia River to practise the ancient craft of swamp rice cultivation, and how, in turn, the human-made changes are exploited by anopheline mosquitoes resulting in increased malaria transmission. This is the first estimate of mosquito production in a rice growing area and confirms our earlier conclusions that rice fields on the edge of the floodplains are a major site for malaria vectors in the middle reaches of the Gambia River [23,24]. These findings suggest that a 100 m strip of rice fields along the edge of the floodplains, 1 km in length, generates 86,500 *An. gambiae *adults/week during the rainy season. This explains why high numbers of vectors can be found in local houses (M. Kirby unpublished data; 64 female *An. gambiae s.l*./house/night, n = 22 houses in the rainy season 2006).

After the seasonal rains start in June, women plough their fields on the landward edge of the floodplains of the Gambia River and prepare raised beds for growing rice there. This is the first area to flood, and by August one set of young plants are transplanted to the fields. Here water is fresh, non-tidal and contains the greatest abundance of insect life on the floodplain. These paddies are clearly demarcated by raised embankments that help keep the fresh water in the fields. However, by October, they are drying out and the second set of young rice plants grown on raised beds near the landward edge are transplanted to fields close to the river from late September to early October. Here the water flows in from the Gambia River; it is salty and tidal, but the fields are flooded and will remain so for several months, long enough for the rice to mature and be cultivated.

It is well-known that rice fields are prolific sources of mosquitoes [4]. Surprizingly, in our study the density of *An. gambiae s.l*. was relatively low with 1.14 larvae/m^2 ^and 0.30 adults/m^2^. Nonetheless the area of rice fields bordering the river is vast, covering many hectares, resulting in prodigious numbers of mosquitoes. In the rice fields, *An. arabiensis *was the most common member of the *An. gambiae *complex. This species is frequently associated with rice fields in The Gambia [23,24] and elsewhere [38-40], although quite why this should be so remains unexplained.

Overall 92% of *An. gambiae *s.l. larvae and 97% of adults came from rice fields close to the landward edge of the floodplains. Where rice is not grown in the same area, this strip of water is likely to be less productive for anophelines due to the lack of impounding. Thus here although short grass is common, the water is tidal and remains for a shorter period. In less tidal parts of the river's floodplain, further up-river, this concentration of vectors in rice fields closer to the villages is less marked, with a greater proportion of vectors emerging further into the floodplain, even close to the river [41].

There are a number of explanations for finding most vectors in rice fields close to the landward edge of the floodplains, related to (1) the geographical position of the fields, (2) their water characteristics, (3) the presence or absence of fish and (4) the concentrations of nutrients.

Rice fields close to the landward edge of the floodplain represent the shortest flight distance for an ovipositing female leaving a village to lay her eggs. Previous studies have demonstrated that rice fields and habitats close to the landward edge were associated with an increased risk of finding anopheline larvae [24,41-43]. The phenomenon of finding larvae on the edges of rice fields has been shown before [44] and other habitats close to human habitation has also been recorded [45,46]. We also found evidence from this study that this effect may exist at a finer spatial scale within individual fields, since higher numbers of larvae were found at the edge closest to the land compared to the edge nearer the river. In common with other studies, larvae were less likely to be found in the centre of the field than on the edge [47].

Rice fields on the landward edge of the floodplain were situated in an area of undisturbed water that was warm, fresh and exposed to sunlight, providing conditions ideal for *An. gambiae s.l*. [22,24]. It was here that most anophelines, culicines and other insect life occurred. The strong positive association between anopheline and culicine larvae has been seen before in The Gambia [24,41,48] and Kenya [8,49]. Nonetheless, it was surprizing to find anophelines sharing habitats with a diverse taxa of insects since many invertebrates are important predators of anopheline [50]. It is possible that the low densities of anophelines can be partly attributed to predation by invertebrate predators. However, in this study fish never occurred where anophelines were found suggesting that fish and mosquitoes are either important predators of mosquitoes [51-54] or that they occupy separate niches. The importance of predators in regulating anopheline populations was impossible to quantify in the present study, and further work is needed to determine which predators feed on the aquatic stages of anophelines in this ecosystem. More details on population dynamics and density of predatory invertebrate taxa in this ecosystem is presented in a separate study [22,24]

Lastly, these findings indicate that cattle dung increases the number of anopheline larvae in breeding sites. This is relevant since during the long hot dry season cattle dung concentrates on the edges of the floodplain when cattle graze on the rice stubble left from the previous season's harvest. In the semi-field trial water with dung was rich in reactive nitrogen and phosphorous, and ammonium radicals. These nutrients are key drivers of invertebrate abundance in aquatic systems [55] and presumably provide the nutrients for the organisms upon which mosquito larvae feed early in the rains. This conclusion is supported by studies in California which found that wetlands rich in ammonium nitrogen had a nine-fold greater mosquito production than wetlands with lower levels of nitrogen [55]. In contrast in Kenya cow dung did not affect larval growth and development [56]. The different findings may result from the fact that double the dose of cow dung was used in the Gambian experiments compared with the Kenyan studies. Clearly the role of cow dung in mosquito productivity merits further study.

The phenology of adult mosquitoes in rice fields has been well documented [10,11,49], but only recently have the larval dynamics been studied [7,40,57]. Findings from the present study concur with previous work showing that larvae colonized rice fields shortly after flooding and remain there until the rice grew tall and/or the fields dried out. Few, if any, larvae are found in mature rice, since vegetation prevents mosquitoes from ovipositing on water [58].

**Figure 6:**
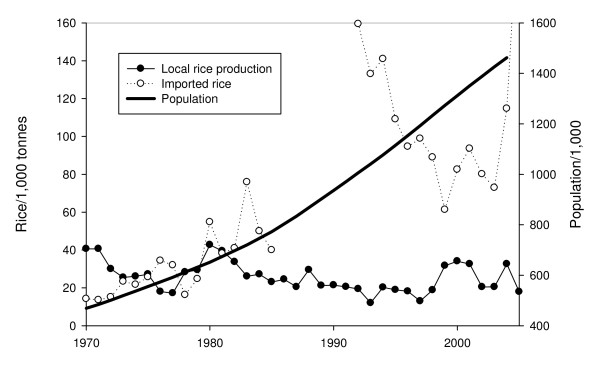
**Local rice production and imports in relation to the rizing population in The Gambia**. No data on imported rice prior to 1990. Data from FAO  and Webb 1992 [21].

Whilst increased production of malaria mosquitoes is an inevitable consequence of rice production, swamp rice cultivation is likely to increase in the future. Rice is the staple food in The Gambia and locally produced rice has failed to keep up with the demand for more rice for the growing population, with imports soaring (Figure 6), straining the country's meagre financial resources. Since the world's consumption of rice outstrips production rice prices are expected to double in the next two years (: accessed 26/6/8). Thus local production of rice must increase, to offset the rapidly increasing cost of imports. Increasing acreages of rice will increase the vector population. Quite what this will mean for the level of malaria in the country is uncertain, since generally in sub-Saharan Africa increasing transmission associated with rice irrigation does not necessarily lead to more malaria [4]. Nevertheless, it is essential to ensure that local communities near rice-growing areas are protected from the potentially lethal infection. This study demonstrates that in this area, treating rice fields close to the landward edge of the floodplains with larvicides would help reduce transmission levels, yet this approach is unlikely to be entirely satisfactory since the extremely low productivity of sites further away will continue to generate appreciable numbers of vectors. In these circumstances attacking the vectors that enter houses with long-lasting impregnated nets, indoor residual spraying or both combined, together with prompt and effective treatment of clinical cases of malaria, should be advocated.

## Competing interests

The authors declare that they have no competing interests.

## Authors' contributions

SWL, UF and LBSJ contributed to study design, data analysis and interpretation of results. CG supervised the PCR analysis and VL conducted the fish sampling aspects of the study. SM supervised the fieldwork. All authors contributed to drafting of the manuscript and all read and approved the final manuscript.
